# Supplementary material for: Partitioning the Effects of Soil Legacy and Pathogen Exposure Determining Soil Suppressiveness via Induced Systemic Resistance
Source: Plants (Basel). 2022 Oct 23;11(21):2816. doi: 10.3390/plants11212816 (PMC9657590; doi:10.3390/plants11212816)
Supplement: Supplementary file 1 [file plants-11-02816-s001.zip › plants-1950702-supplementary.pdf]

Article title: Partitioning the effects of soil legacy and pathogen exposure determining soil suppressiveness via induced systemic resistance

Authors: Na Zhang, Chengzhi Zhu, Zongzhuan Shen, Chengyuan Tao, Yannan Ou, Rong Li, Xuhui Deng , Qirong Shen, Francisco Dini-Andreote

Supplementary Files:

Table S1. General information of the sequencing data

**Sample**	**Clean Reads No.**	**Clean Data (bp)**	**Clean Reads %**	**Clean Data %**
EBF-Rs1	36025014	5354674526	98.51	96.97
EBF-Rs2	44102074	6403135174	98.34	94.55
EBF-Rs3	41636146	6162210472	98.4	96.45
ECF-Rs1	41493732	6153607750	98.4	96.64
ECF-Rs2	40453744	6025452764	98.36	97.02
ECF-Rs3	42440208	6253260840	98.42	96.03

Note: Sample：Sample name;

Clean Reads No.: High quality sequence read number;

Clean Data (bp): High quality sequence base;

Clean Reads %: Percentage of high-quality reads in sequenced reads;

Clean Data %: Percentage of high quality sequence bases in sequencing bases.

EBF-Rs: rhizosphere or root derived from the BF soil and plants were set to grow in the split-root system for 7 days prior to the introduction of *R. solanacearum* into the soil; ECF-Rs: rhizosphere or root derived from the CF soil and plants were set to grow in the split-root system for 7 days prior to the introduction of *R. solanacearum* into the soil.

Table S2. RNA-Seq Map

Sample	Useful Reads	Map Events count	Total Mapped Reads	Total Mapped %	Multiple Mapped Reads	Multiple Mapped %	Uniquely Mapped Reads	Uniquely Mapped %
EBF-RsL1	360250	30932094	30512409	84.70	288830	0.95	30223579	99.05
EBF-RsL2	441020	39070491	38478409	87.25	356371	0.93	38122038	99.07
EBF-RsL3	416361	33348339	32780420	78.73	384240	1.17	32396180	98.83
ECF-RsL1	414937	35483436	34883643	84.07	438341	1.26	34445302	98.74
ECF-RsL2	404537	34561338	34071861	84.22	296633	0.87	33775228	99.13
ECF-RsL3	424402	35606689	35036399	82.55	362927	1.04	34673472	98.96

Note: EBF-Rs: rhizosphere or root derived from the BF soil and plants were set to grow in the split-root system for 7 days prior to the introduction of *R. solanacearum* into the soil; ECF-Rs: rhizosphere or root derived from the CF soil and plants were set to grow in the split-root system for 7 days prior to the introduction of *R. solanacearum* into the soil.

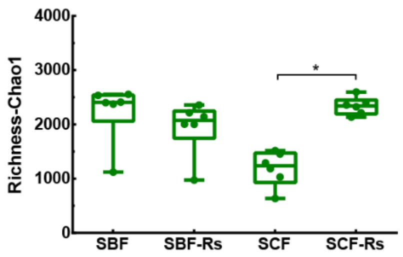

Figure. S1 *Alpha* diversity analysis of rhizosphere bacterial communities (Chao1) in Experiment I. The asterisks indicate significant differences between treatments determined by two sample *t*-tests (* P < 0.05). SBF: rhizosphere derived from the BF soil without *R solanacearum* inoculation; SCF: rhizosphere derived from the CF soil without *R solanacearum* inoculation; SBF-Rs: rhizosphere derived from the BF soil and inoculated with *R. solanacearum* at the same time that plants were transferred to the split root system; SCF-Rs: rhizosphere derived from the CF soil and inoculated with *R. solanacearum* at the same time that plants were transferred to the split root system.

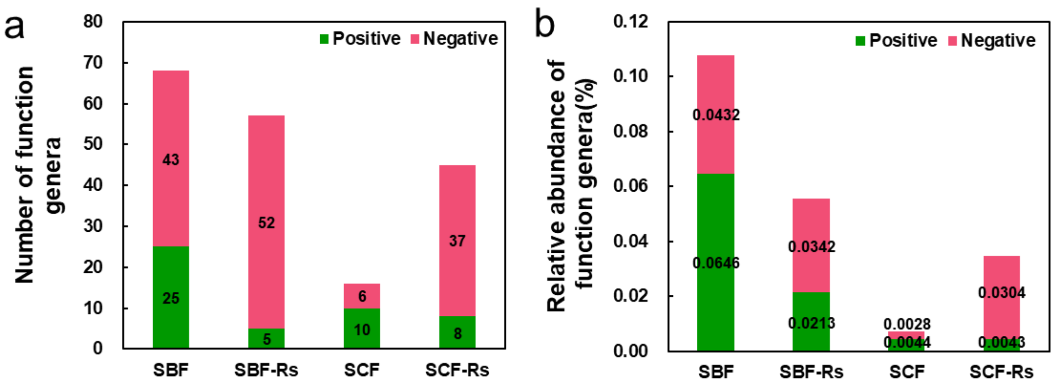

Figure. S2 Responsive bacterial taxa in the Experiment I. The number (**a**) and relative abundance (**b**) of responsive bacterial genera. SBF: rhizosphere derived from the BF soil without *R solanacearum* inoculation; SCF: rhizosphere derived from the CF soil without *R solanacearum* inoculation; SBF-Rs: rhizosphere derived from the BF soil and inoculated with *R. solanacearum* at the same time that plants were transferred to the split root system; SCF-Rs: rhizosphere derived from the CF soil and inoculated with *R. solanacearum* at the same time that plants were transferred to the split root system.

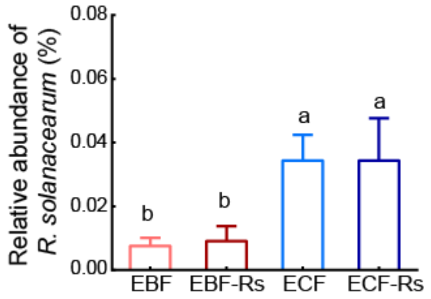

Figure. S3 Relative abundance (16S sequencing) of *R. solanacearum* in Experiment II. Different letters above the bars indicate significant differences at $P < 0.05$ based on one-way analysis of variance (ANOVA). EBF: rhizosphere derived from the BF soil without *R solanacearum* inoculation; ECF: rhizosphere derived from the CF soil without *R solanacearum* inoculation; EBF-Rs: rhizosphere or root derived from the BF soil and plants were set to grow in the split-root system for 7 days prior to the introduction of *R. solanacearum* into the soil; ECF-Rs: rhizosphere or root derived from the CF soil and plants were set to grow in the split-root system for 7 days prior to the introduction of *R. solanacearum* into the soil.

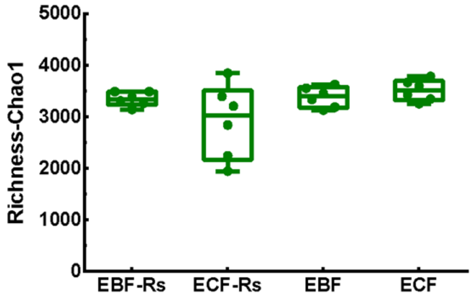

Figure. S4 *Alpha* diversity analysis of the rhizosphere bacterial communities (Chao1) in Experiment II. EBF: rhizosphere derived from the BF soil without *R solanacearum* inoculation; ECF: rhizosphere derived from the CF soil without *R solanacearum* inoculation; EBF-Rs: rhizosphere or root derived from the BF soil and plants were set to grow in the split-root system for 7 days prior to the introduction of *R. solanacearum* into the soil; ECF-Rs: rhizosphere or root

derived from the CF soil and plants were set to grow in the split-root system for 7 days prior to the introduction of *R. solanacearum* into the soil.

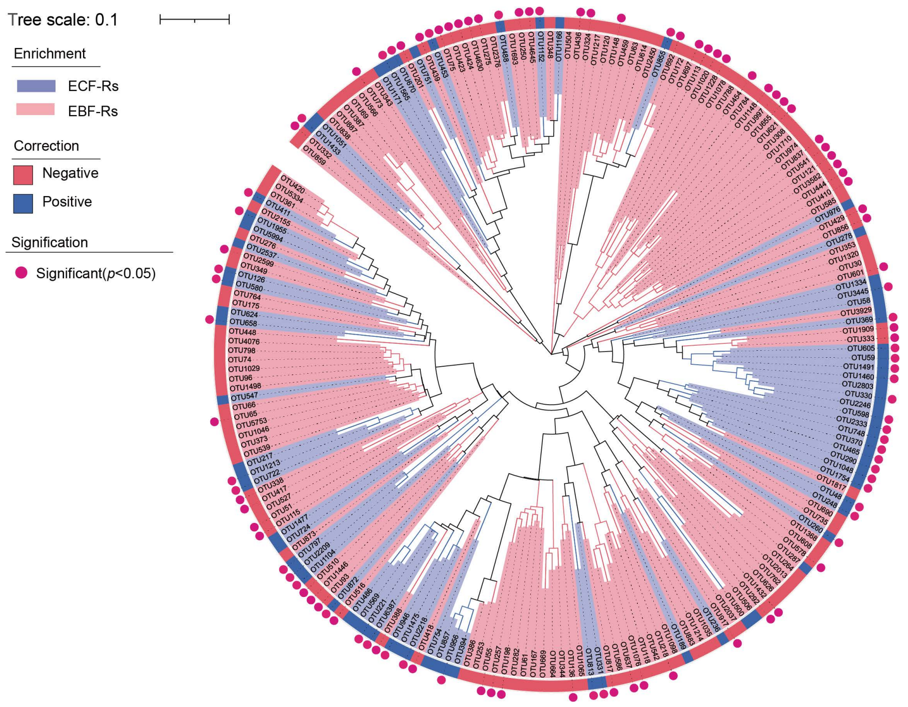

Figure. S5 Spearman's rank correlation analysis between significantly different OTUs between treatments with pathogen inoculation (EBF-Rs vs ECF-Rs) in Experiment II. EBF-Rs: rhizosphere or root derived from the BF soil and plants were set to grow in the split-root system for 7 days prior to the introduction of *R. solanacearum* into the soil; ECF-Rs: rhizosphere or root derived from the CF soil and plants were set to grow in the split-root system for 7 days prior to the introduction of *R. solanacearum* into the soil.

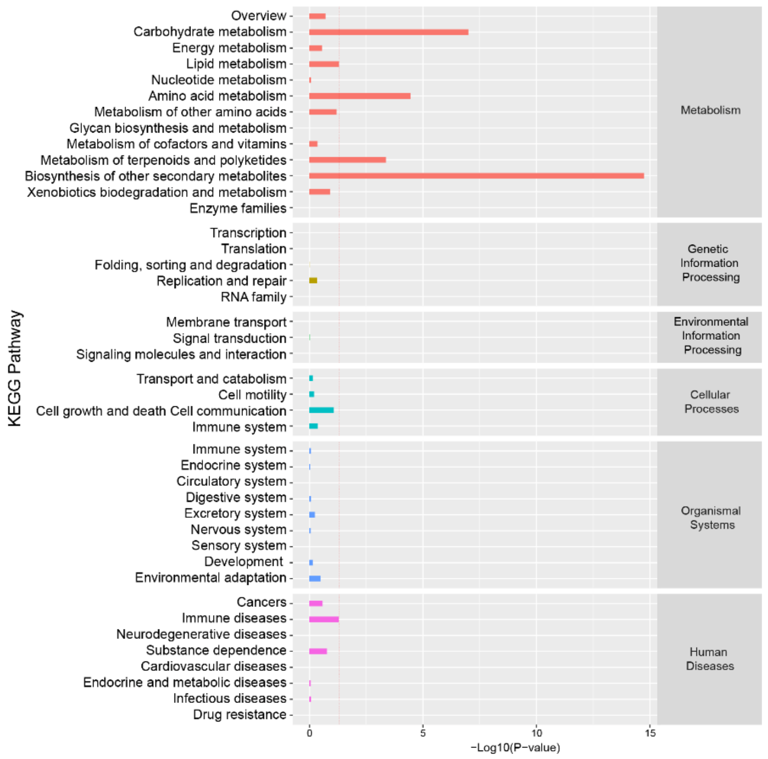

Figure. S6 Overall data based on KEGG analysis.

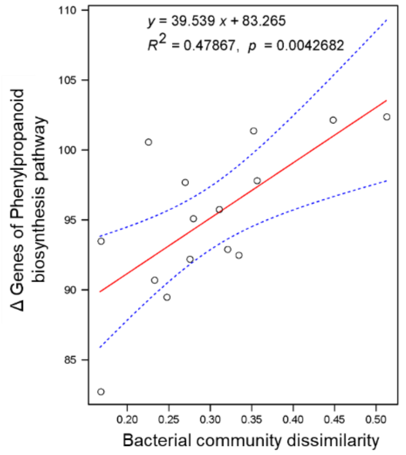

Figure. S7 Mantel test between bacterial communities and the Phenylpropanoid biosynthesis pathway.
